# What Is a Working Equid? Analysis of Current Terminology and a Suggested Definition

**DOI:** 10.3390/ani14142026

**Published:** 2024-07-09

**Authors:** Zoe Raw, Joseph A. Collins, Faith A. Burden

**Affiliations:** 1 Bristol Veterinary School, Langford House, Dolberry, Bristol BS40 5DU, UK; 2 The Donkey Sanctuary Ireland, Knockardbane, Mallow, P51 PH29 Cork, Ireland; joe.collins@thedonkeysanctuary.ie; 3 The Donkey Sanctuary, Slade House Farm, Weston, Sidmouth EX10 0NU, UK; faith.burden@thedonkeysanctuary.org.uk

**Keywords:** working equid, terminology, low- and middle-income countries, high-income countries, literature review, definitions, nomenclature

## Abstract

**Simple Summary:**

Equids, such as horses, donkeys, and mules, play vital roles in various types of work globally, with their use differing significantly across countries of different income levels. We conducted a thorough review of research literature to examine if and how terms related to working equids are used in high-, middle-, and low-income countries. Our findings indicate that studies focusing on higher-income countries tend to produce more research papers on equids, except for when using the terms “working equid” and “draft equid”, which are more common in studies focusing on lower-income countries, and these terms often refer to animals in low-resource rural settings and marginalised communities. We suggest that “working equid” should be defined as “any equid engaged in physical labour that provides a significant or direct contribution to the economic livelihood, sustenance or support of the owner/user’s family, typically within a low resource setting”. The lack of a standard definition complicates policy making and welfare legislation, especially in lower-income countries where equids are crucial for livelihoods but are often overlooked by policy. This study highlights the need for more inclusive and culturally aware terminology to improve welfare standards and policies for working equids worldwide.

**Abstract:**

Equids are engaged in myriad types of work across the world, with contexts and industries varying widely between high-, middle- and low-income countries as classified by the World Bank. Through a comprehensive abstract search and literature review, we examined the usage and context of terms associated with working equids in high-income countries (HICs), upper-middle-income countries (UMICs), lower-middle-income countries (LMICs), and low-income countries (LICs). Analysis showed that the search term used was significantly associated with World Bank country income classification. All search terms except two returned a significantly higher number of papers for higher-income countries compared to lower-income countries. The two exceptions were “working equid” and “draft equid”, which returned significantly more papers for lower-income countries than higher-income countries. Both terms also returned papers from high-income and upper-middle-income countries, but these were always in the context of low-resource settings and rural or marginalised communities, of which we provide examples in the discussion. We propose that the term “working equid” should be used to describe “any equid engaged in physical labour that provides a significant or direct contribution to the economic livelihood, sustenance or support of the owner/user’s family, typically within a low resource setting”. Our review highlights the intricate interplay between socioeconomic factors and examines how equids are described in the literature. The lack of a universally accepted definition leads to challenges in policy formulation, resource allocation, and welfare standards development, particularly in LMICs and LICs where working equids are crucial to livelihoods. This study underscores the need for a more inclusive and comprehensive approach to terminology, advocating for future research to bridge linguistic and cultural gaps in understanding working equids globally. Such efforts are vital for equitable and effective welfare standards and policy development for these animals.

## 1. Introduction

Recognition of the value of consistent terminology in research is not new; in fact, most modern science is built on foundations that first sought to classify and define concepts in the world around us, for example, taxonomy and binomial nomenclature [1,2], chemistry and pharmacology [3,4], disease diagnosis and genetics [5,6,7], physics and astronomy [8,9]. It is perhaps surprising, therefore, that only recently has the importance of consistent nomenclature in animal sciences emerged as a topic of value; work has shown that the irregular use of correct names confounds the conservation of both wild and feral animal populations [10,11,12,13,14] and the lack of consistent terminology is shown to cause confusion in the scientific literature across behavioural and evolutionary sciences [15]. Research has sought to define terms used for companion and “therapy” animals [16], but this has not yet been extended to the field of animals engaged in economic activity or labour.

### 1.1. The Case of Working Equids

Equids (horses, donkeys, and their hybrids) serve as critical assets across various global economies. Their roles are multifaceted, and they are used for a variety of purposes in different countries, including transport [17,18,19,20,21], domestic work [22,23,24,25], agriculture [21,26,27,28,29,30], forestry [31,32,33,34], sports [35], racing [36], leisure [37,38], tourism [39], ceremonial duties [40,41,42], and production [43,44,45]. Despite their myriad uses, however, those that are considered to be “working” are variously manifested throughout different economic classifications, with distinct differences between lower- and higher-income countries [26].

In lower-income settings, working equids often function as the backbone of rural economies [46,47]. They are frequently employed in agriculture for ploughing fields, sowing seeds, and transporting harvests. They perform household chores and contribute to rural livelihoods by transporting people, water, food, firewood to the homestead, and goods for sale to the market. Working equids also provide a supply of natural fertiliser and, in some cases, milk, meat, and hides for their owners or users [48]. Beyond agriculture and domestic use, working equids are integral to transportation, particularly in remote areas where roads are underdeveloped and motorised vehicles are scarce, for example, in mountainous regions [49,50]. They have been shown to play an important role in disaster relief [51,52] and, in settings where mechanisation is limited or absent, equids become indispensable, and reliance upon them is absolute. They are also used as a work tool by marginalised communities; for instance, they play an integral role in the brick kilns throughout India and Nepal [18,23,53,54,55,56].

Working equids in lower-income settings are typically found in rural and marginalised communities, and they often exhibit poor welfare, attributed to owners’ limited financial resources due to poverty or unstable living conditions or limited physical resources to meet the animals’ basic needs such as adequate nutrition, access to water, veterinary care, or a suitable living environment [17,19,23,46,57]. The owners of working equids in lower-income settings are typically without formal education and live in poverty, struggling with significant humanitarian challenges, unemployment, or geopolitical instability [24,53,58,59,60]. Despite the significant contributions working equids make to household and national economies in such settings [26,61,62], there is often little formal recognition of this contribution, and working equids are considered to be under-represented and under-considered at national and global policy levels [26,63].

Conversely, in higher-income economies, the role of equids is considerably more individually specialised and often less essential for supporting basic livelihoods since they mainly fulfil sports, racing, leisure, tourism, and recreation roles [64,65]. However, many of the equids in such economies could be considered as “working” since they generate (sometimes substantial) revenue for their owners and trainers [66,67]. For instance, horse racing and polo are sports that generate income and support multi-million-pound industries [68,69]. For context, the combined sports horse, racing, and leisure breeding industry across Europe is valued at EUR 52.1 billion per annum [70]. Equids engaged in sports, racing, competition, and leisure might be defined as “working” since they “… perform physical or mental labour; to exert oneself for a definite purpose, esp. in order to produce something or to earn a living…” [71]. Unlike the owners of working equids in lower-income contexts, the owners of sports horses and equine athletes in richer economies tend to have high levels of formal education and possess relatively large disposable incomes [72,73,74].

In addition to high-value equine industries in higher-income countries, equids are also still engaged in working in more traditional roles, i.e., they perform physical work (traction and load carrying) in service of people and communities. Specific examples include the equids used for tourism in Santorini, Greece [75], equids used for forestry throughout Europe [31,34], equids used for milk production [43,44], and equids used to draw tourist carriages in many European cities, the operation of which provides the equid owner with a livelihood [76,77]. The continued use of equids in agricultural and manual work in higher-income countries is due to a variety of reasons, including that terrain makes it impossible to use motorised vehicles, so equid power is still used, or in rural communities where they maintain or want to resurrect a more traditional way of life [47,78]. Higher-income countries usually benefit from more advanced and robust frameworks to protect and prioritise equid welfare, but this is not without its own set of challenges and criticisms [79,80].

### 1.2. Statement of the Problem: Lack of Clear, Universally Accepted Definitions

It is clear, therefore, that working equids contribute significantly to global and national economies across countries of all economic classifications and in a variety of settings. However, the term “working equid” has become synonymous with describing horses, donkeys, and their hybrids engaged in physical work across lower-income countries, particularly in the Global South [81], and the term is generally associated with some of the most resource-limited and vulnerable communities in the world [22,47,61,82,83]. In such a context, it is widely reported that there are an estimated 112 million working horses, donkeys, and hybrids worldwide, which support the livelihoods of marginalised or rural communities [84]. However, as we have established, equids that perform work in higher-income settings and the Global North [85] also contribute significant economic sums to both their industry and their owners. Surprisingly, the literature does not classify these equids as ”working equids”; rather, they are typically described as sports horses, racehorses, competition horses, polo ponies, equine athletes, and leisure horses [86,87,88].

One of the most critical yet understated issues in the discourse surrounding working equids is the absence of clear, universally accepted definitions. Despite extensive research and multiple publications in the field, the terminology associated with working equids often remains ambiguous, inconsistent, and sometimes contradictory [22,23,59,89]. This terminological nebulousness extends from academic literature to policy documents, hindering effective communication among researchers, policymakers, and practitioners. Firstly, the lack of a standard definition for the term “working equid” creates a systemic problem for data collection and analysis. Traditional livestock censuses and studies are generally framed around established categories of animals. However, equids typically fall into a grey zone, neither strictly categorised as companion animals, pets, or leisure animals, but yet also not defined as conventional livestock like cattle or poultry [56,90]. This results in a data gap that leaves working equids as an overlooked sector of the global animal population [90,91], which in turn affects their prominence or acknowledgement in terms of resource allocation, planning, and policy formulation, particularly in lower resource settings where the importance of working equids is more critical for human and community survival [83,92].

Second, the absence of clear definitions makes it challenging to assess welfare indicators effectively. Different studies and projects use differing criteria to categorise working equids, ranging from their functional roles (e.g., transport, agriculture) to specific physiological and behavioural markers. This inconsistency muddles comparative studies and meta-analyses, thereby impacting the quality of research and its applicability in real-world settings [93,94,95,96]. Third, the lack of standardised terminology leads to a disjointed legislative and regulatory landscape: the use of consistent terminology is recognised as a key element to progress in other fields [97,98,99]. With no universally accepted definitions, laws, or regulations of working equids, the welfare, use, and trade of working equids can differ vastly between jurisdictions, both within and across countries [48,80].

This lack of synchrony complicates the efforts of the major international non-governmental organisations (INGOs) working in this field. In 2017, the International Coalition for Working Equids (ICWE) was formed specifically with the aim of presenting a unified voice for the four major UK-based organisations working in this area, namely Brooke, SPANA, The Donkey Sanctuary, and World Horse Welfare, which aim to harmonise practices and standards across regions [26,58]. The ICWE has become an important voice for working equids across the world and has worked with the World Organisation for Animal Health (WOAH, founded as OIE) to support the implementation of the working equid standards, detailed in Chapter 7.12 of the Terrestrial Animal Health Code [48]. The ICWE has positioned itself as a champion for improved working equid welfare: it provides expert advice during disease outbreaks, gives technical support to governments, and creates tools and resources for communities and equid owners. The unified position of a group such as the ICWE and the collective progress that comes from a single consolidated voice serve as an important reminder of why the use of consistent terminology is so important.

The implications of this problem are not merely academic but have real-world consequences for the welfare of working equids and the communities that depend on them. The lack of clarity in defining what constitutes a “working equid” contributes to gaps in our understanding, thereby influencing the focus and effectiveness of interventions aimed at improving their welfare [23,55,57,58,94]. Using consistent terminology in global animal health initiatives is crucial for effective collaboration and multi-stakeholder communication. Using a common language streamlines programme planning and execution, simplifies resource allocation, and enables smoother international partnerships. It aids in monitoring and evaluation, allowing teams to quickly assess a project’s success or identify areas for improvement. Consistency in terminology also facilitates clear public communication, garnering broader support for initiatives [100]. Standardising the terms used by NGOs working in this area is a necessary prerequisite to more accurate data, improved comparative studies, streamlined regulations, and, ultimately, enhanced animal welfare and human livelihoods.

### 1.3. Purpose of This Study and Research Questions

While the significance of working equids in all economies is well-established, there remains a significant gap in how these animals are defined and categorised across academic disciplines, policy documents, and among practitioners. Such inconsistencies result in challenges ranging from effective data collection to the execution of welfare programmes. The overarching aim of this study is to review the literature to understand the current use of some key terms and present a suggested definition as to how the term “working equid” is currently being used.

This study aims to analyse and quantify whether different definitions relating to working equids are used in different economic settings, and we expect that different search terms will be associated with different economic contexts. The results will be discussed in the context of how standardised definitions are used and applied in research. The need for such standardisation in the field of working equid welfare has been raised previously but has yet to be comprehensively addressed [22,23,24,59,82,95,101]. These questions will support a wider discussion about how standardised terminology could influence policy formulation, resource allocation, and public awareness, thus directly impacting the welfare of these animals and their human caretakers [22,23,56,60]. Given the interdisciplinary nature of this subject—spanning veterinary science, economics, and social sciences—this study could serve as a foundational piece for future research and policy interventions.

## 2. Methods

### 2.1. Search Terms

We used bibliometric analysis to search the literature to understand how different search terms might be associated with different income contexts. Following discussions with experts in the equestrian scientific research community and feedback from reviewers, we selected fifteen search terms associated with equids in work. The search terms used are presented in Table 1; “intended target/definition” refers to the colloquial use of the search term, and the “actual search term used” refers to the words plus Boolean operator(s) used in the database search. Henceforth, any reference to “search term” will relate to the intended target/definition given in Table 1.

### 2.2. Literature Search

We used the terms in Table 1 to perform a literature search for peer-reviewed papers on the Scopus database. We initially also searched PubMed and Web of Science but found the results to be the same for Scopus, so we limited our search to Scopus alone. Google Scholar was excluded due to its tendency to include grey literature and non-peer-reviewed sources [102]. Additionally, reports from international non-government organisations (INGOs) were also included if they were publicly accessible on Scopus. For each of the search terms used, we categorised the results by (i) country of focus, (ii) World Bank country income classification (CIC), and (iii) the context or setting within which the equids were working.

### 2.3. Inclusion/Exclusion Criteria

Articles were included if they were written in English, were peer-reviewed, and were related to equids. We did not set a date range, and articles were included regardless of age. Articles were excluded if they did not focus on equids or were not in English.

## 3. Analysis

### 3.1. Country of Focus

Once a search was complete, the search data were extracted from Scopus as a .csv file and imported into Excel for analysis. Scopus provides a ‘Country’ listing for each paper, but manual review revealed that these classifications were misleading; papers tended to be attributed to the lead author’s country of institutional affiliation and not the location studied in the paper. Therefore, we manually reviewed each paper and changed the country of focus to the country where the fieldwork was carried out to generate more accurate information on the actual setting of the equids being studied.

### 3.2. Income Classification

Once the search results had been correctly defined by country of focus (i.e., study site location), we used the current World Bank guidelines to classify countries as either low-income countries (LICs), lower-middle-income countries (LMICs), upper-middle-income countries (UMICs), or high-income countries (HICs). The World Bank country income classification (CIC) method calculates each country’s gross national income (GNI) per capita as the dollar value of a country’s final income in a year divided by its population using Atlas methodology [103]. The most recent (2022) definitions are as follows: (i) LICs are those with a GNI per capita of USD 1085 or less; (ii) LMICs have a GNI per capita between USD 1086 and USD 4255; (iii) UMICs are those with a GNI per capita between USD 4256 and USD 13,205; and (iv) HICs have a GNI per capita of USD 13,205 or more [104].

Once we had defined the country of focus and the World Bank CIC, this enabled us to see the number of papers that were returned for each search term, disaggregated by World Bank CIC. “Number of papers” was used as a proxy to start exploring how commonly each search term is used to describe working equids across different economic contexts. In addition, we found that some papers focused on LMIC or LIC settings in general and not a particular study site, so we created an additional category of “General LMIC/LIC” to better reflect the broader nature of these examples.

### 3.3. Statistical Analysis

Once the CIC of each country had been defined, we counted the total number of papers returned for each search term for each of the four economic classifications (LIC, LMIC, UMIC, HIC) plus the new ‘General LMIC/LIC’ category. Search terms with too few or irrelevant results were discarded (see results). For the remaining search terms, a multinomial logistic regression analysis was conducted in SPSS to examine the relationship between each of the search terms and the number of papers returned relating to each World Bank CIC plus the new General LMIC/LIC category. The dependent variable (CIC) had five categories: LIC, LMIC, UMIC, HIC, and General LMIC/LIC. The independent variables were the search term (categorical with eight levels) and the number of papers found per country (continuous). To further understand how individual search terms were associated with high or low CICs, we conducted a Kruskal–Wallis *H* test for each search term with post hoc pairwise comparisons to identify the difference in the number of papers returned for each CIC.

## 4. Results

### 4.1. Number of Papers Returned per Search Term

All search terms yielded generally applicable results, with a few exceptions. “Working equi*” returned results about “working equipment”, so we changed the search term to “Working equi*” AND NOT “working equipment”, which solved the problem. Likewise, “packhorse” yielded results about “packhorse lobsters”, so we changed the search term to “packhorse* NOT lobster”, which then returned only studies focused on equids. The number of papers returned for each of the fifteen search terms and their disaggregation by CICs are listed in Table 2.

Of the fifteen search terms used (Table 1), seven were excluded from any further analysis due to either (i) too few search results (leisure equid, pack donkey, pack equid, pack pony, sports pony, working pony) or (ii) results relating to historical accounts (pack horse), which are not directly relevant to today’s discourse.

### 4.2. Multinomial Logistic Regression Analysis

The overall model was statistically significant, *χ*^2^ = 83.23 [28], *p* < 0.001, indicating that the World Bank CIC could be significantly predicted by the search terms based on the number of papers returned per CIC. The model explained 68.5% (Nagelkerke R^2^) of the variance, and likelihood-ratio tests confirmed that ‘search term’ was the significant predicting factor in the model (*p* < 0.001). The parameter estimates for the multinomial logistic regression model predicting CIC based on the search term and the number of papers are presented in Table 3.

### 4.3. Analysis by Search Term

Draft equid: There was a significant difference in the number of papers returned across country income classifications; H(2) = 8.725, *p* = 0.013. There were significantly more papers for LMICs (mean = 7.50) when compared to HICs (m = 1; *p* = 0.009). There was no significant difference between the number of papers returned between LMICs and UMICs (m = 2; *p* = 0.162) or between UMICs and HICs (*p* = 1.000).

Draft horse: There was no significant difference in the number of papers returned across country income classifications; H(3) = 7.168, *p* = 0.067.

Equine athlete: There was a significant difference in the number of papers returned across country income classifications; H(2) = 8.261, *p* = 0.016. There were significantly more papers for HICs (m = 13.10) compared to both UMICs (m = 1.25; *p* = 0.033) and LMICs (m = 1.00; *p* = 0.28). There was no significant difference between UMICs and LMICs.

Leisure horse: There was a significant difference in the number of papers returned across country income classifications; H(2) = 4.798, *p* = 0.009. There were significantly more papers for HICs (m = 4.77) compared to both UMICs (m = 1.00; *p* = 0.004) and LMICs (m = 1.00; *p* = 0.04). There was no significant difference between UMICs and LMICs.

Racehorse: There was a significant difference in the number of papers returned across country income classifications; H(3) = 23.008, *p* < 0.001. There were significantly more papers for HICs (m = 58.65) compared to UMICs (m = 7.89; *p* = 0.026), LMICs (m = 2.14; *p* = 0.006) and LICs (m = 1.00; *p* = 0.000). There was no significant difference between UMICs, LMICs, and LICs.

Sports horse: There was a significant difference in the number of papers returned across country income classifications; H(2) = 4.615, *p* < 0.001. There were significantly more papers for HICs (m = 26.96) compared to UMICs (m = 11.33; *p* = 0.026) and LMICs (m = 9.00; *p* = 0.004), and significantly more papers for UMICs compared to LMICs (*p* < 0.001).

Working equid: There was a significant difference in the number of papers returned across country income classifications; H(4) = 9.734, *p* = 0.002. Significantly more papers were returned for LMICs (m = 6.58) compared to HICs (m = 9; *p* < 0.001), UMICs (m = 3.4; *p* < 0.001), LICs (m = 14.33; *p* = 0.001), and General LMIC/LIC (m = 36; *p* = 0.004). A further *t*-test showed that there were significantly more papers for lower-income categories (i.e., LMICs, LICs, and General LMIC/LICs combined) than for higher-income categories (UMICs and HICs combined); *t*(28) = 4.502, *p* < 0.001.

Working horse: There was no significant difference in the number of papers returned across country income classifications; H(4) = 3.111, *p* = 0.540.

## 5. Discussion

We investigated a variety of search terms relating to working equids to establish whether particular terms have become synonymous with different settings and if this might be related to the World Bank CIC [104]. Of fifteen representative search terms, seven were found to be irrelevant or little used, but the remaining eight search terms returned relevant papers. We found that World Bank CIC could be significantly predicted by the search terms based on the number of papers returned per CIC, supporting our initial hypothesis that different search terms would be associated with different economic contexts.

Our analysis showed that all search terms, except two, returned progressively fewer papers as CIC reduced. That is, when HICs were used as the reference category, stepwise comparisons through reducing CICs (i.e., from HICs > UMICs > LMICs > LICs > General LMIC/LIC) showed a decreasing number of papers. This indicates that all analysed search terms (except two) generally returned a higher number of papers for countries classified as higher income, whether that be in a HIC or UMIC setting. For instance, both “Equine athlete” and “Leisure horse” showed a significant decrease in stepwise comparisons from HICs to UMICs and from UMICs to LMICs, indicating that these terms are used more predominantly in higher-income countries. The search for “Equine athlete” returned just one paper relating to an LMIC and none for LICs or General LMIC/LICs. Likewise, the search for “Leisure horse” returned just two papers relating to an LMIC and none for LICs or General LMIC/LICs. The term “Racehorse” returned similar results, with just one paper returned for LICs and 30 for LMICs, with a much higher return rate for papers relating to UMICs and HICs (*n* = 2488).

The first exception to this trend was the search term “working equid”; this search term returned progressively more papers in downward stepwise comparisons from HICs through to LICs, i.e., the use of this term increased as country income decreased. This search term yielded significantly more papers in downward stepwise comparisons from HICs to UMICs and from UMICs to LMICs. The comparison between LMICs and LICs was non-significant, but this could be explained by the very high number of results that were attributed to General LMIC/LICs. We then combined the General LMIC/LICs category with the LIC data and found that there were significantly more papers returned for ‘lower-income’ categories (i.e., LMICs, LICs, and General LMIC/LICs combined) than for higher-income categories (UMICs and HICs combined). The second exception was the search term “Draft equid”; this search term returned more papers for LMICs (*n* = 15) than for both UMICs and HICs combined (*n* = 13), demonstrating that its use increased as country income decreased.

Although our results show that the term “working equid” is significantly associated with lower-income countries (LMICs and LICs), we found that it returned papers for some UMICs (Mexico, Brazil, Colombia, Argentina, Cuba, and Ecuador) and some HICs (Portugal, Uruguay, Chile, and Spain). This could be attributed to several factors, including income inequality between regions and the existence of traditional rural communities within these countries. Despite the overall wealth of HICs and UMICs, not all regions or populations within them share equally in this prosperity, and there are often underprivileged and marginalised communities where the use of working equids persists either due to economic necessity or sometimes in relation to preserving a more traditional way of life [105,106,107,108,109]. Working equids are well documented to be integral to the livelihoods of such communities, performing agricultural, transportation, and other utilitarian roles that mechanisation has not replaced due to financial constraints or cultural preferences [19,47,57]. Furthermore, in HICs, there can be a deliberate choice to maintain traditional methods for ecological reasons, such as reducing fossil fuel dependence or due to environmental fragility and the preservation of traditional and historic farming practices [32,33,110]. These factors can contribute to an intentional use of working equids, aligning with a global rise in sustainability efforts [26,111,112].

All the papers we reviewed that related to higher-income countries were focused on rural or marginalised communities following a subsistence or more traditional way of life. For instance, the papers returned for Portugal and Spain (both HICs) interviewed “60 working equid owners from rural communities”, and ninety-five percent of participants “were farmers involved in small-scale agriculture. Equids were used for ploughing land, sowing, and harvesting potatoes. Many of the participants were subsistence farmers, with the vegetables grown feeding them throughout the year”. The study participants spoke about “the importance and utility of donkeys for people’s everyday life”, “One participant did not know how they would work without the help of their equid [“She helps a lot. If it was not for the animal what would I do?”], and those who could not afford access to other methods carried out all farming work with equids” [47]. A further study reported that working equids in Spain are used by “…rural and often remote [communities], with high migration of younger generations to urban areas, leaving an older population maintaining a traditional agricultural way of life” [113]. This supports our observation that despite a country being classified as high-income, the role of working equids is confined to localised, low-income settings that are typically marginalised or rural communities maintaining a traditional way of life, where equids are used to support the owner’s economic livelihood or sustenance.

Papers relating to “working equids” in UMICs highlighted the following statements about the communities studied: in Mexico, “[communities were] characterised as of high marginality and social poverty”, “…where farmers rely on working equines for agricultural activities” [114]; in Brazil, “In rural areas, equids play an important economic and social role, through performing haulage work. The health of these animals and their physical performance are crucial for income generation, since these animals are the basis of financial support for countless families” [115]; in Colombia, “two-thirds of [working equid] owners in Colombia [are] living below the International Poverty Line with an income less than USD 1.90 a day” [116]; in Argentina, the working equid’s main role “includes transportation (riding, pack transport, or cart pulling), being its use for entertainment or as a source of food” [117]; in Cuba, “Horses are mainly used for transport, including important urban transport services in several cities. Mules are used for packing in the hills, and donkeys are maintained to breed mules” [118]; and in Ecuador, “…donkey use has declined since the era of mechanization and modern farming practices, but the animals are still used in rural areas for traditional roles such as transportation of agricultural products, firewood and water, riding, and, in some urban areas, although to a lesser extent, to pull carts” [33].

### 5.1. The Case for a Universal Definition

The discourse surrounding working equids is complex and embedded in diverse cultural, economic, and social practices globally [26,49,52,53,54,119]. The absence of a standardised definition for “working equids” presents a multi-layered issue that hampers not only academic discourse but also the development and implementation of policies aimed at improving the welfare of these animals. Here, we present our rationale for establishing a universal definition, providing a clearer framework for research, policy making, and welfare initiatives. First, the lack of a standardised definition creates significant challenges for ensuring and protecting animal welfare [120,121,122,123]. Without a clear definition, it is difficult to develop and enforce regulations that protect the wellbeing of working equids [48]. This lack of clarity often results in inadequate legal protections, leaving these animals vulnerable to overwork, neglect, and abuse [61,91]. A universal definition would facilitate the creation of targeted welfare standards and improve the effectiveness of monitoring and enforcement mechanisms, ensuring a consistent level of care [89,124]. Second, a standardised definition is critical for comparative research. Inconsistencies in terminology can lead to difficulties in comparing studies across different geographical regions, cultures, and subjects [125], potentially skewing data and impeding our understanding of global trends [22,23,58,95,96]. Standardised terminology would enable researchers to aggregate data more accurately, providing a more reliable evidence base to inform policy and practice.

Third, the lack of a universal definition affects the allocation of resources and funding; organisations that support working equids often rely on data to prioritise their efforts, so ambiguity in definitions can lead to the misallocation of resources or to certain populations of working equids being overlooked entirely [53,55,56,58]. A clear definition would aid these organisations in identifying the most pressing needs and in tracking the impact of their interventions more effectively. Fourth, in the context of international development, a universal definition is a minimum standard expected by global agencies [12,97,98,99]. The role of working equids in livelihoods varies significantly between HICs and LICs, yet the contributions they make are often undervalued due to definitional ambiguity [25]. Recognising working equids as essential components of the agricultural workforce in many parts of the world could elevate their status within development agendas, ensuring they receive appropriate consideration in planning and resource allocation [21,26,52,119]. Finally, standardising the definition of working equids is not only a scientific and economic imperative but also a moral one; it reflects a commitment to equity in animal welfare and recognises the intrinsic value of these animals beyond their utilitarian roles.

### 5.2. A Standard Definition

Our research suggests that the use of the term “working equids” in the literature is characterised by the following attributes: (i) that the equid performs tasks that require physical exertion, such as pulling agricultural equipment, a cart, carrying crops or water canisters, or transporting people and other goods; (ii) that the tasks these equids perform contribute directly to the owner/user’s economic livelihood, sustenance or ability to support their family; and (iii) that they are working in a low-resource setting, but not necessarily in a low-income country. The third point, therefore, does not restrict the term “working equid” to LMICs and LICs and instead allows for cases where equids are used for manual tasks in subsistence or marginalised communities within UMICs or HICs.

Having identified these three important facets of the way “working equid” is currently used, we propose that the term “working equid” should be defined as “any equid engaged in physical labour that provides a significant or direct contribution to the economic livelihood, sustenance or support of the owner/user’s family, typically within a low resource setting”. This definition, built upon the three facets observed in the literature, further functions to support the exclusion of racehorses, sports horses, and equine athletes in this definition. Although it is true that these animals engage in physical exertion for their owner/user, and it could be argued that they have a significant or direct influence on the owner’s economic position, they are not operating within low-resource settings.

Since this study is about the importance of using consistent definitions, it is worth noting that throughout the literature, the acronym LMIC is often incorrectly and broadly used to describe “low- and middle-income countries” when it is actually defined by the World Bank as referring to “lower-middle-income countries” (LMICs) with “low-income countries” (LICs) being a separate category altogether [104]. Organisations and investigators should be careful to refer to “LICs and LMICs” and not just “LMICs” if they are referring to low- and middle-income countries.

### 5.3. Limitations and Future Directions

We recognise that this study focused on a small number of search terms, but the study was designed to be an initial exploration of definitions and to stimulate further discussion about how definitions are used in the global equid welfare field. We suggest that future research could use a wider range of terms to analyse how they are used. In addition, our analysis was restricted to reliance on existing literature, which is inherently biased towards English language publications, which may preclude access to studies centred on LICs and LMICs. There may also be a bias towards papers from HICs and UMICs being carried out and published due to the availability of funding in such countries compared to LMICs and LICs. Another limitation is the methodology of literature search and selection, which, despite being thorough, might have inadvertently excluded relevant grey literature or unpublished studies that could offer additional insights [126]. It is significant to consider that much information about working equids in rural or underserved areas might not be formally published, but this dovetails with existing discourse about how to make research more culturally inclusive and representative of all cultures [127,128,129,130]. Furthermore, this study’s focus on academic and policy documents might not fully reflect the terms’ usage in everyday practice by equid owners, veterinarians, and local communities. This observation highlights the need for future research involving field studies and qualitative methods such as interviews and ethnographic work to capture the lived experiences and terminologies used within and between communities and countries [131,132].

Future research could focus on including a broader range of languages and sources, including non-academic literature and oral histories, to enrich the understanding of working equid terminology. Collaborative international efforts involving researchers from a variety of countries, especially LMICs and LICs, could provide a more comprehensive perspective [133,134]. There is also a need for longitudinal studies to understand how the terminology and the roles of working equids evolve over time, particularly in response to changing economic, environmental, and social factors [21,125,135]. Finally, interdisciplinary research that combines perspectives from veterinary science, anthropology, economics, and sociology could provide a more holistic view of the role and perception of working equids in different societies.

## 6. Conclusions

This study has demonstrated that the term “working equid” is not necessarily restricted to countries classified as low income, yet is used in reference to low resource contexts, where the owner or user typically relies on that equid to sustain a livelihood, sustenance, or support their family. Additionally, we have identified the urgent need for a universally accepted definition of the various different types of all “working” equids to bridge the disparity in terminology across economic boundaries. Future research should encompass a broader array of linguistic and cultural contexts, aiming to integrate local insights into global academic and policy discussions. Ultimately, developing a unified, inclusive terminology is essential for equitable welfare standards and effective policy making for working equids worldwide.

## Figures and Tables

**Table 1:** The fifteen search terms associated with working equids used to identify their common use in the scientific literature.

Intended Target/Definition	Actual Search Term Used
Draft equid/equine or draught equid/equine	“Draft equi*” OR “Draught equi*”
Draft horse or draught horse	“Draft horse*” OR “Draught horse*”
Equine athlete	“Equi* athlete*”
Leisure equid/equine	“Leisure equi*”
Leisure horse	“Leisure horse*”
Pack donkey	“Pack donk*”
Pack equid/equine	“Pack equi*”
Pack horse	“Packhorse*” OR Pack horse*”
Pack pony	“Pack pon*”
Racehorse	“Racehorse*”
Sports horse	“Sport* horse*”
Sports pony	“Sport* pon*”
Working equid/equine	“Working equi*”
Working horse	“Working horse*”
Working pony	“Working pon*”

**Table 2:** The number of papers returned for each of the fifteen search terms, plus disaggregation by World Bank country income classification, plus the additional General LMIC/LIC category.

Search Term	Total Papers	HICs	UMICs	LMICs	LICs	General LMIC
Draft equid/equine or draught equid/equine	28	*n* = 7 (25.0%)	*n* = 6 (21.4%)	*n* = 15 (53.6%)	*n* = 0	*n* = 0
Draft horse or draught horse	407	*n* = 378 (92.9%)	*n* = 12 (2.9%)	*n* = 14 (3.4%)	*n* = 3 (0.7%)	*n* = 0
Equine athlete	386	*n* = 380 (98.4%)	*n* = 5 (1.3%)	*n* = 1 (0.3%)	*n* = 0	*n* = 0
Leisure equid/equine	0	*n* = 0	*n* = 0	*n* = 0	*n* = 0	*n* = 0
Leisure horse	110	*n* = 105 (95.5%)	*n* = 3 (2.7%)	*n* = 2 (1.8%)	*n* = 0	*n* = 0
Pack donkey	12	*n* = 0	*n* = 0	*n* = 11 (91.7%)	*n* = 1 (8.3%)	*n* = 0
Pack equid/equine	3	*n* = 0	*n* = 2 (66.7%)	*n* = 1 (33.3%)	*n* = 0	*n* = 0
Pack horse *	44	*	*	*	*	*n* = 0
Pack pony	0	*n* = 0	*n* = 0	*n* = 0	*n* = 0	*n* = 0
Racehorse	2519	*n* = 2346 (93.1%)	*n* = 142 (5.6%)	*n* = 30 (1.2%)	*n* = 1 (0.1%)	*n* = 0
Sports horse	866	*n* = 755 (87.21%)	*n* = 102 (11.8%)	*n* = 9 (1.0%)	*n* = 0	*n* = 0
Sports pony	8	*n* = 8 (100%)	*n* = 0	*n* = 0	*n* = 0	*n* = 0
Working equid/equine	219	*n* = 27 (12.3%)	*n* = 34 (15.5%)	*n* = 79 (36.1%)	*n* = 43 (19.6%)	*n* = 36 (16.4%)
Working horse	170	*n* = 80 (47.1%)	*n* = 32 (18.8%)	*n* = 37 (21.8%)	*n* = 14 (8.2%)	*n* = 7 (4.1%)
Working pony	2	*n* = 0	*n* = 2	*n* = 0	*n* = 0	*n* = 0

* “Pack horse” returned 44 papers, but these were all related to discussions about history, so they were discounted as they are not directly relevant to the current use of terminology.

**Table 3:** Parameter estimates for the multinomial logistic regression model predicting country income classification based on search term and number of papers.

Income Classification ^a^							
UMIC							
	Predictor	β	SE	Wald χ^2^	df	*p*	Exp(B)
	Intercept	0.052	0.433	0.014	1	0.095	
	No of papers	−0.068	0.021	10.631	1	**0.001**	0.934
	Draft equid	−0.741	0.812	0.833	1	0.362	0.477
	Draft horse	−1.205	0.653	3.406	1	0.065	0.300
	Equine athlete	−1.612	0.685	5.532	1	**0.019**	0.200
	Leisure horse	−1.813	0.750	5.848	1	**0.016**	0.163
	Racehorse	−0.027	0.527	0.003	1	0.959	0.973
	Sports horse	−0.159	0.603	0.070	1	0.792	0.853
	Working equid	1.747	0.803	4.740	1	**0.029**	5.740
	Working horse	0 ^b^			0		
LMIC							
	Intercept	0.169	0.447	0.143	1	0.706	
	No of papers	−0.098	0.034	8.325	1	**0.004**	0.907
	Draft equid	−1.203	0.913	1.739	1	0.187	0.300
	Draft horse	−1.697	0.751	5.102	1	**0.024**	0.183
	Equine athlete	−2.995	1.106	7.331	1	**0.007**	0.050
	Leisure horse	−2.261	0.857	6.964	1	**0.008**	0.104
	Racehorse	−0.258	0.545	0.225	1	0.635	0.772
	Sports horse	−2.249	1.122	4.018	1	**0.045**	0.105
	Working equid	1.984	0.802	6.127	1	**0.013**	7.272
	Working horse	0 ^b^			0		
LIC							
	Intercept	−1.286	0.671	3.672	1	0.055	
	No of papers	−0.037	0.043	0.760	1	0.383	0.963
	Draft equid	−17.574	4923.971	0.000	1	0.997	2.331 × 10^−8^
	Draft horse	−0.967	0.982	0.969	1	0.325	0.380
	Equine athlete	−17.873	3078.518	0.000	1	0.995	1.729 × 10^−8^
	Leisure horse	−17.938	3416.049	0.000	1	0.996	1.621 × 10^−8^
	Racehorse	−1.789	1.216	2.164	1	0.141	0.167
	Sports horse	−17.239	2834.112	0.000	1	0.995	3.261 × 10^−8^
	Working equid	1.656	1.060	2.441	1	0.118	5.239
	Working horse	0 ^b^			0		
General LMIC							
	Intercept	−3.862	1.598	5.840	1	**0.016**	
	No of papers	0.173	0.121	2.042	1	0.153	1.189
	Draft equid	−17.123	0.000	0.000	1	0.938	3.662 × 10^−8^
	Draft horse	−25.276	1758.302	0.000	1	0.989	1.053 × 10^−11^
	Equine athlete	−33.818	451.883	0.006	1	0.940	2.057 × 10^−15^
	Leisure horse	−18.871	5745.069	0.000	1	0.997	6.375 × 10^−9^
	Racehorse	−129.587	1667.187	0.006	1	0.938	5.261 × 10^−57^
	Sports horse	−27.619	2089.479	0.000	1	0.989	1.012 × 10^−12^
	Working equid	−1.374	3.098	0.197	1	0.657	0.253
	Working horse	0 ^b^			0		

^a^ The reference category is HIC. ^b^ This parameter is set to zero because it is redundant. Significant results are indicated in bold.

## Data Availability

The primary data are available in Table 2.
